# Impaired fatigue resistance, sarcoplasmic reticulum function, and mitochondrial activity in soleus muscle of db/db mice

**DOI:** 10.14814/phy2.15478

**Published:** 2022-09-18

**Authors:** Hiro Yamamoto, Hiroaki Eshima, Saori Kakehi, Ryuzo Kawamori, Hirotaka Watada, Yoshifumi Tamura

**Affiliations:** ^1^ Department of International Tourism Nagasaki International University Nagasaki Japan; ^2^ Department of Metabolism & Endocrinology Juntendo University Graduate School of Medicine Tokyo Japan; ^3^ Sportology Center Juntendo University Graduate School of Medicine Tokyo Japan; ^4^ Center for Therapeutic Innovations in Diabetes Juntendo University Graduate School of Medicine Tokyo Japan; ^5^ Center for Identification of Diabetic Therapeutic Targets Juntendo University Graduate School of Medicine Tokyo Japan

**Keywords:** calcium, contractile function, diabetes, mitochondria, sarcoplasmic reticulum, skeletal muscle, slow twitch muscle

## Abstract

Type 2 diabetes mellitus (T2DM) is characterized by reduced exercise tolerance due to increased fatigability in skeletal muscle. In this study, we investigated muscle fatigue resistance of soleus (SOL) muscle in obese type 2 diabetic model mice (db/db). No differences in muscle volume, absolute force, or specific force in SOL muscle were observed between db/db mice and control mice (db/+), while fatigue resistance evaluated by repeated tetanic contractions was significantly lower in db/db mice (30th tetani, db/+: 63.7 ± 4.7%, db/db: 51.3 ± 4.8%). The protein abundance related to Ca^2+^ release from the sarcoplasmic reticulum (SR) in SOL muscle was not different between db/db mice and db/+ mice, while SR Ca^2+^‐ATPase (Ca^2+^ reuptake to SR) protein was decreased in db/db mice compared to db/+ mice (db/+: 1.00 ± 0.17, db/db: 0.60 ± 0.04, relative units). In addition, mitochondrial oxidative enzyme activity (succinate dehydrogenase) was decreased in the SOL muscle of db/db mice (*p* < 0.05). These data suggest that fatigue resistance in slow‐twitch dominant muscle is impaired in mice with T2DM. Decreased mitochondrial oxidative enzyme activity and impairment of Ca^2+^ uptake to SR, or both might be involved in the mechanisms.

## Graphical abstract

The present study demonstrated decreased fatigue resistance in slow‐twitch dominant muscle from db/db mice. Decreased oxidative enzyme activity of mitochondria, and impairment of Ca^2+^ handling of SR, or both might be involved in the underlying mechanisms. Our data may provide a basis for the reduced fatigue resistance during high‐intensity strength training exercises for Type 2 diabetic patients.

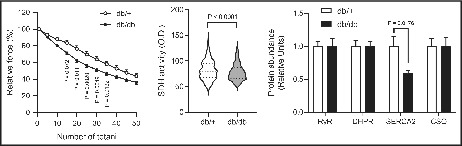

## 1 INTRODUCTION

Type 2 diabetes mellitus (T2DM) is characterized by reduced exercise tolerance due to increased fatigability in skeletal muscle (Nesti et al., 2020; Wei et al., 2000). This phenomenon may be induced by impaired cellular functions, such as Ca^2+^ release‐reuptake by the sarcoplasmic reticulum (SR), mitochondrial energy supply, and/or muscle fiber type composition (Eshima et al., 2014; Schiaffino & Reggiani, 2011). Previous studies demonstrated that type 2 diabetes impairs oxygen supply within muscle microcirculation (Padilla et al., 2006, 2007). A previous study showed that the mitochondrial respiratory capacity was decreased in the skeletal muscle of T2DM patients (Kelley et al., 2002). In addition, we recently identified reduced fatigue resistance and intracellular Ca^2+^ dysregulation together with dysfunction of SR in an animal model of T2DM (Eshima et al., 2019). Those studies focused on fast‐twitch dominant muscles because diabetes induces fiber‐type‐specific effects predominantly targeting fast‐twitch skeletal muscle (Wang & Pessin, 2013). Indeed, the percentage of myosin heavy chain (MHC) type IIb fibers in skeletal muscle is higher in patients with T2DM than in control subjects (Yasuda et al., 2006) and the greater physiological fragility of fast‐twitch fibers than slow‐twitch fibers was observed in type 1 diabetic rats (Eshima et al., 2015). However, no studies have focused on decreased fatigue resistance in slow‐twitch skeletal muscle of individuals with T2DM.

Based on this background, we investigated muscle fatigue resistance and the underlying mechanisms of slow‐twitch dominant muscle fibers in db/db mice, an obese T2DM model mouse. We tested the hypotheses that fatigability is exacerbated in the slow‐twitch dominant muscle of db/db mice, and this phenomenon is related to organelle dysfunction, such as SR and mitochondrial oxidative enzyme activity.

## 2 METHOD

### 2.1 Animals

Twelve‐week‐old db/db (C57BL/KsJ‐lepr^db^/lepr^db^; db/db) male mice and age‐matched controls heterozygote (db/+) male mice were purchased from CLEA. The db/db mice (*n* = 20) and db/+ mice (*n* = 20) in this study were from a previous study (Eshima et al., 2019). We confirmed that the body mass was significantly higher in db/db (46.4 ± 0.9 g) mice than in db/+ mice (28.6 ± 0.4 g; *p* < 0.0001). The mice were maintained under a 12:12‐h light–dark cycle with ad libitum access to food and water. All experiments were conducted under the guidelines established by the Physiological Society of Japan and were approved by the Animal Experimental Committee of Juntendo University.

### 2.2 Muscle preparation

Soleus (SOL) muscles were used for all experiments, including contraction measurements, histochemical staining, and Western blotting. Mice were anesthetized via intraperitoneal injection of sodium pentobarbital (70 mg/kg body wt), and their muscles were dissected once a surgical level of anesthesia was reached, after which tissues were harvested. Muscle preparations consisting of intact SOL samples were assessed, as described previously (Eshima et al., 2021).

### 2.3 Contraction measurement

The force–frequency curve was assessed in ex vivo SOL muscle as previously described (Eshima et al., 2021). Briefly, isolated SOL muscle preparations were mounted between a force transducer (UL‐100; Minebea Co.) and a fixed hook in a chamber containing Krebs solution (120 mmol/L NaCl, 5 mmol/L KCl, 2 mmol/L CaCl_2_, 1 mmol/L MgCl_2_, 1 mmol/L NaH_2_PO_4_, 25 mmol/L NaHCO_3_, and 11 mmol/L glucose) bubbled with 95% O_2_ and 5% CO_2_ at 30°C. The optimal length was determined by the maximum twitch force. Next, the isolated muscle was stimulated with 500‐ms trains of pulsed current at 10, 20, 30, 40, 50, 70, 100, or 150 Hz at 1‐min intervals, and contractile force was measured. After muscle length was measured, the muscles were removed from the chamber, the tendons were dissected, and muscle mass was measured. The specific force was calculated from the absolute force, muscle mass, and muscle length, assuming a density of 1.056 g/mL. Fatigue was subsequently induced by 50 tetanic stimulations for 500 ms at 100 Hz separated by 2‐s intervals.

### 2.4 Muscle histology

Mouse muscle fibers in the histological sections were examined and analyzed as described previously (Eshima, Tamura, et al., 2017). Briefly, serial 10‐μm sections were cut with a cryostat (CM1510; Leica) at −20°C and mounted on polylysine‐coated slides. Succinate dehydrogenase (SDH) activity in individual muscle fibers in the histological sections was examined and analyzed as previously described (Eshima et al., 2013, 2015). Whole sections were stained for slow and fast MHCs. Mouse monoclonal antibodies that react specifically with type I (BF‐F3), type IIa (SC‐71), or type IIx (BF‐35) MHC isoforms were supplied by the Developmental Studies Hybridoma Bank (University of Iowa, IA, United States). The M.O.M. Immunodetection kit (Vector Laboratories) and Vectastain ABC kit (Vector Laboratories) were used to assess immunohistochemical reactions according to the manufacturer's instructions. SDH activity was recorded using a camera (E1000M; Nikon, Japan) at ×10 magnification and subsequently analyzed subsequently in ImageJ (NIH). The cross‐sectional areas and SDH activities were measured by tracing fiber outlines of ∼160 fibers from the muscle sections of any individual mouse. The images were digitized as gray‐level pictures. Each pixel was quantified as one of 256 gray levels and then automatically converted to optical density using ImageJ software. With the use of those gray‐level pictures, the SDH activity of any individual muscle fibers was quantified using the forbidden line rule (Baum et al., 2016).

### 2.5 Western blot analysis

SR‐related proteins were analyzed by Western blotting as previously described (Eshima, Tamura, et al., 2017). The protein abundance of the ryanodine receptor (RyR), the dihydropyridine (DHPR), the calsequestrin (CSQ), and the SR Ca^2+^‐ATPase (SERCA) was assessed. Briefly, polyvinylidene fluoride membranes were incubated overnight at 4°C with the following primary antibodies: anti‐type 1 ryanodine receptor (RyR) antibody 34C (MA3‐925; Thermo Fisher Scientific); anti‐dihydropyridine (DHPR) antibody 20A (ab2864; Abcam); anti‐calsequestrin antibody VIIID12 (MA3‐913; Thermo Scientific); anti‐SR Ca^2+^‐ATPase 2 (SERCA2) antibody 2A7‐A1 (MA3‐919; Thermo Scientific), and anti‐ glyceraldehyde‐3‐phosphate dehydrogenase (GAPDH) antibody 14C10 (no. 2118; Cell Signaling Technology) at 4°C. The membranes were incubated with the appropriate secondary antibody conjugated to horseradish peroxidase, enhanced by SuperSignal West Dura and Femto extended duration substrate (Thermo Fisher Scientific), and quantified by densitometry (C‐DiGit, LI‐COR Biosciences).

### 2.6 Statistical analysis

Values are expressed as means ± SE. Statistical analyses were performed with Prism version 9.0 (GraphPad Software). When two nonpaired groups were compared, Student's *t*‐test was applied. For comparisons of groups, two‐way ANOVA with a Tukey's post‐hoc test was used. For all tests, *p* < 0.05 was considered statistically significant.

## 3 RESULTS

**FIGURE 1:**
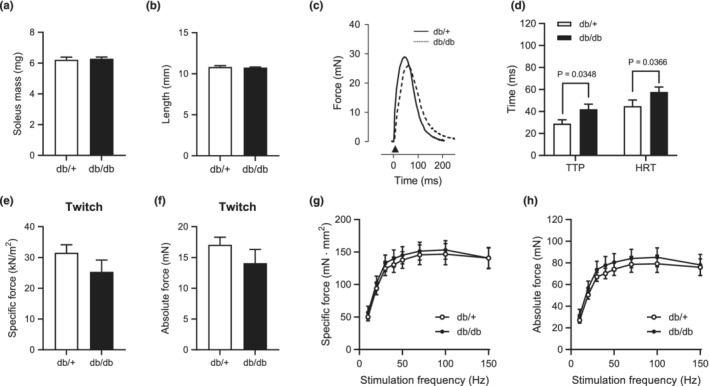
Contractile properties in soleus muscles of db/db mice. (a) and (b): SOL muscle mass (a) and muscle length (b). (c): Representative twitch force tracings in SOL muscle. (d): TTP and HRT of twitches. (e) and (f): Specific (e) and absolute (f) peak twitch forces for SOL muscles. (f) and (g): Force‐frequency curve in SOL muscles of db/db mice. The presented data are the means ± SE (*n* = 7 per group).

Muscles from db/+ mice and db/db mice did not differ in SOL muscle mass, length, or twitch in either specific force or absolute force (Figure 1a,b,c,e,f). The time to peak force (TTP) and the half‐relaxation time (HRT) under twitch force were prolonged in db/db mice compared with that of db/+ mice (Figure 1d). However, there was no difference in the force–frequency curve in either specific force or absolute force between db/+ mice and db/db mice (Figure 1g,h). These data suggest that SOL muscle of db/db mice maintains the contractile force but shows latent contractile abnormalities.

**FIGURE 2:**
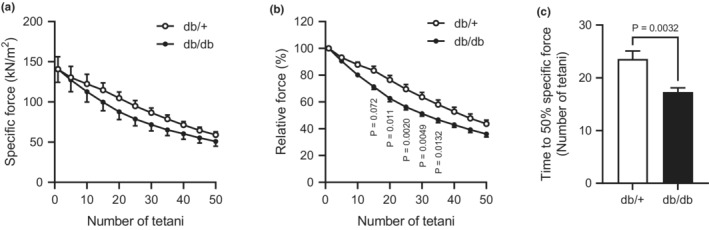
Fatigue resistance with repeated tetanic stimulation in SOL muscle of db/db mice. (a): Absolute specific force decay during repetitive high‐frequency stimulation (500 ms, 100 Hz, and 2‐s interval). (b): Normalized specific force decay during repetitive high‐frequency stimulation. (c): Time to 50% specific force. A two‐way ANOVA with post hoc analysis including Tukey's multiple comparisons were used (a and b). An unpaired two‐tailed *t*‐test was used (c). The presented data are the means ± SE (*n* = 7 per group).

In the fatigue experiments, the tetanic force decreased more rapidly in both the normalized‐specific force and absolute force in SOL muscle of the db/db mice than in that of db/+ mice (Figure 2a,b). At the time to 50% specific force decreased in db/db compared to db/+ (Figure 2c). These data indicate that fatigability during tetanic contraction is increased in slow‐twitch muscles of db/db mice compared with those of db/+ mice.

**FIGURE 3:**
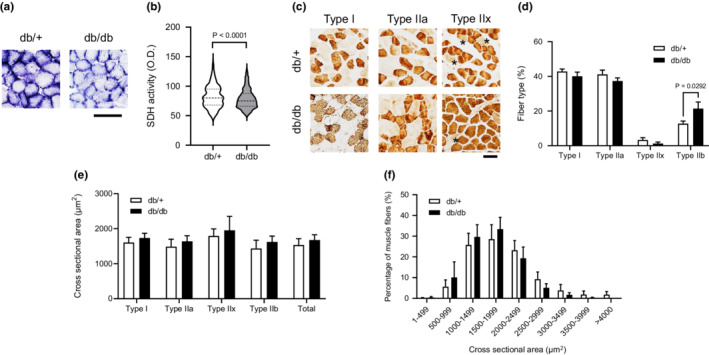
Morphological characterization and mitochondrial oxidative capacity of SOL muscle in db/db mice. (a): Transverse SOL muscle sections stained for SDH activity. Scale bar = 50 μm. (b): Quantification of SDH activity of any individual fibers (db/+, *n* = 797; db/db, *n* = 782). (c): Transverse SOL muscle sections stained for MHCs antibody. Scale bar = 50 μm. *MHC type IIx fibers. (d): Fiber‐type composition for SOL muscles. (e): Muscle fiber CSA by fiber type for SOL muscles. (f): Distribution of muscle fiber CSA for soleus muscles. An unpaired two‐tailed *t*‐test was used (b). A two‐way ANOVA with post hoc analysis including Tukey's multiple comparisons were used (d–f). The presented data are the means ± SE (*n* = 5 per group).

Next, we investigated the muscle morphology and mitochondrial oxidative enzyme activity in SOL muscles of db/db mice. SDH activity was decreased in db/db mice compared with db/+ mice (Figure 3a,b). The percentage of MHC type IIb fibers was increased in db/db mice compared with db/+ mice (Figure 3c,d). We did not find any differences in cross‐sectional area (CSA) in either the frequency histogram analysis or the fiber type analysis between the groups (Figure 3e,f).

**FIGURE 4:**
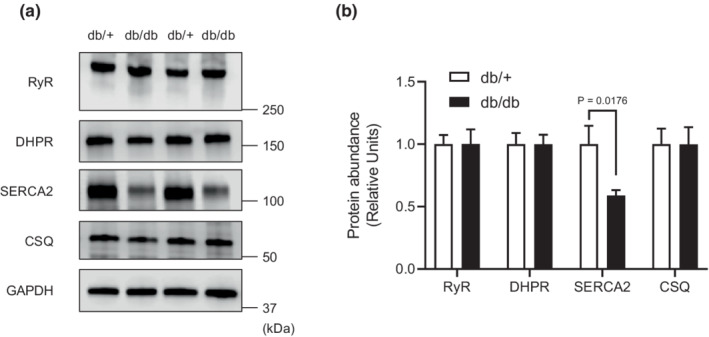
Abundance of calcium‐regulated proteins. Representative Western blots are shown of RyR, DHPR, SERCA2, CSQ, and GAPDH protein abundance in SOL muscle. The presented data are the means ± SE (*n* = 8 per group).

Furthermore, we evaluated critical proteins involved in calcium regulation in skeletal muscle. The protein abundance of RyR, DHPR, and CSQ was comparable between db/+ mice and db/db mice. Intriguingly, the protein abundance of SERCA2, which is responsible for cytosolic Ca^2+^ uptake into SR, was significantly lower in db/db mice than in db/+ mice (Figure 4a,b).

## 4 DISCUSSION

In this study, we investigated whether T2DM accelerates the fatigability of a slow‐twitch dominant muscle. We found that decreased fatigue resistance was evaluated by repeated tetanic contraction in SOL muscle of db/db mice, while the contractile force was maintained. In addition, db/db mice have lower mitochondrial oxidative enzyme activity and Ca^2+^‐ATPase protein content in SOL muscle than the control mice.

Reduced contractile force production in skeletal muscle of type 2 diabetes may be partly explained by diabetic motor neuropathy (Andersen et al., 1997). Indeed, contractile force by nerve stimulation was decreased in the soleus muscle of db/db (Bayley et al., 2016). However, our data showed isolated muscle contraction force ex vivo by electrical stimulation was maintained in the soleus muscle of db/db (Figure 1e,f). The reason for different results maybe different stimulation protocols and or influenced by the motor neuron system. On the other hand, we found decreased fatigue resistance in SOL muscle of db/db mice, a rodent model for obesity and type 2 diabetes; however, it remains unclear whether obesity or diabetes was the cause of the decreased fatigue resistance. A previous study showed that T2DM patients have decreased contractile power in lower‐limb muscle during fatigue experiments due to alters contractile properties, suggesting increased fatigability of skeletal muscle in T2DM (Senefeld et al., 2018). In terms of obese type 2 diabetic animals, db/db mice exhibited impaired fatigue resistance in fast‐twitch skeletal muscle (Eshima et al., 2019; Ostler et al., 2014). However, fatigue resistance in skeletal muscle was not altered (Farkas et al., 1994; Shortreed et al., 2009), elevated (Eshima et al., 2021), or decreased (Warmington et al., 2000) in nondiabetic obese model mice. The reason for the different results is unclear, but models of genetic obesity or diabetes may have a difference in muscle fiber size and function in several skeletal muscles (Krause et al., 2009; Ostler et al., 2014). Here, the present study suggests that the decrease in the fatigue resistance of skeletal muscle by db/db mice may, in part, be attributed to a decrease in the fatigue resistance of skeletal muscle by T2DM, not obesity.

Numerous studies using rodent animals have demonstrated that rodents with diabetes have decreased fiber CSA and impaired force production in fast twitch fibers, whereas slow‐twitch fibers are affected to a lesser extent or not at all (Eshima et al., 2014; Ostler et al., 2014). Consistently, no significant change was found in muscle mass, length, or CSA in soleus muscle from db/db mice (Figures 1a,b and 3e,f). Indeed, db/db mice had maintained contractile function in slow‐twitch muscle in the present study (Figure 1g,h). However, db/db mice had a higher proportion of type IIb MHC fibers in SOL muscle (Figure 3d). Consistently, in genetic type 2 diabetic rodent models [e.g., Goto‐Kakizaki (GK) rats or Otsuka Long‐Evans Tokushima Fatty (OLETF) rats], a slow‐to‐fast transition of MHC isoforms in skeletal muscle has been observed (Yasuda et al., 2002, 2006). Thus, it is likely that altered fiber type composition is caused by diabetes. The type IIb contains a comparatively low density of mitochondria and has lower resistance to fatigue compared to type I/IIa (Schiaffino & Reggiani, 2011), suggesting that altered fiber type may be involved in decreased fatigue resistance in slow‐twitch dominant muscle of db/db.

We observed decreased mitochondrial oxidative enzyme (SDH) activity in db/db mice compared to healthy mice (Figure 3a,b). Similarly, a previous study showed the smaller mitochondrial size and decreased electron transport chain activity in the muscle mitochondrial fractions of insulin‐resistant rodent models (Bonnard et al., 2008) and patients with obesity and type 2 diabetes (Ritov et al., 2005). In addition, it has been shown that patients with type 2 diabetes have decreased mitochondrial oxidative phosphorylation activity (Petersen et al., 2005) (Lowell & Shulman, 2005). A previous study showed that both Citrate Synthase (CS) and SDH activity were decreased in the skeletal muscle of GK rats (Lai et al., 2017). We only measured SDH activity in slow‐twitch muscle of db/db, but it may be also a decrease in mitochondrial function such as CS activity in db/db. Because exercise capacity and fatigue resistance depend on the proportion of oxidative fiber and mitochondrial content in skeletal muscle (Heden et al., 2019), T2DM lowers fatigue resistance in slow‐twitch muscle due to decreases in the muscle mitochondrial content and function.

One putative mechanism for functional alterations in diabetic skeletal muscle could be impairments of Ca^2+^ release and uptake from SR (Eshima, 2021). A previous study demonstrated that genetically induced nondiabetic obese mice showed impaired Ca^2+^ handling in skeletal muscle fibers (Bruton et al., 2002). Indeed, we recently demonstrated a substantial degree of impairment in [Ca^2+^]_i_ homeostasis in skeletal muscle of db/db mice, suggesting that decreased Ca^2+^ release may contribute to skeletal muscle contractile dysfunction in db/db mice. These observations have been shown in rodent animal models using the flexor digitorum brevis (FDB) muscle, a fast‐twitch dominant muscle (Ainbinder et al., 2015). We found no change in Ca^2+^ release‐related protein (RyR, DHPR, and CSQ) abundance in slow‐twitch dominant muscle of db/db mice, which may be linked to maintained contractile force in slow‐twitch dominant muscle. In contrast, db/db mice had decreased SERCA2 protein abundance (Figure 4b), and this observation was consistent with a previous study showing reduced SERCA content in db/db mice (Bayley et al., 2016). In addition, a previous study indicated that higher TTP and HRT (Figure 1d) are caused by impaired Ca^2+^ release and uptake from the SR of muscle (Eshima et al., 2019). Allen and colleagues proposed that a fatigued state is associated with dysfunction of Ca^2+^ uptake into SR (Allen et al., 2008) and, therefore, causes accumulation of intracellular Ca^2+^ in skeletal muscle (Eshima et al., 2013). Indeed, a previous study using heart muscle of db/db showed decreased SERCA activity (Belke et al., 2004), which may be soleus muscle also causing reduce SERCA activity. Thus, the decrease in fatigue resistance in db/db mice might be partly due to impaired Ca^2+^ flux via decreased SERCA content in slow‐twitch dominant muscle. A previous study demonstrated that SR‐mitochondria interactions are reduced in obesity and T2DM (Tubbs et al., 2018). Indeed, calcium regulation by mitochondria may be important in fatigue resistance in skeletal muscle (Eshima, Miura, et al., 2017).

The present study has a number of limitations. First, sample numbers were small, increasing the sample size would increase the statistical power of the analysis. Second, the present study also used only male mice similar to the previous report (Eshima et al., 2019). Fatigue properties and SERCA activity in skeletal muscle in males are different from those in females (Harmer et al., 2014); thus our data may not be generalized to the female model. Third, the relationship between fatigue resistance and Ca^2+^ handling or mitochondrial function in the slow‐twitch dominant muscle of db/db mice is still unclear, and further investigations are needed.

In conclusion, this study demonstrated decreased fatigue resistance in slow‐twitch dominant muscle from db/db mice. Decreased oxidative enzyme activity of mitochondria, and impairment of Ca^2+^ handling of SR, or both might be involved in the underlying mechanisms. Our data may provide a basis for reduced fatigue resistance during high‐intensity strength training exercises for Type 2 diabetic patients.

## AUTHOR CONTRIBUTIONS

Hiroaki Eshima conceived and designed research; Hiroaki Eshima performed experiments; Hiro Yamamoto and Hiroaki Eshima analyzed data; Hiro Yamamoto and Hiroaki Eshima interpreted results of experiments; Hiro Yamamoto and Hiroaki Eshima prepared figures; Hiro Yamamoto, Hiroaki Eshima, and Yoshifumi Tamura drafted the manuscript; Hiro Yamamoto, Hiroaki Eshima, Saori Kakehi, Ryuzo Kawamori, Hirotaka Watada, and Yoshifumi Tamura, approved the final version of the manuscript.

## FUNDING INFORMATION

This article was supported by the KAKENHI from the Ministry of Education, Culture, Sports, Science, and Technology of Japan.

## CONFLICT OF INTEREST

The author declares that the research was conducted in the absence of any commercial or financial relationships that could be construed as a potential conflict of interest.
